# Heterogeneity of Pulmonary Granulomas in Cattle Experimentally Infected With *Mycobacterium bovis*

**DOI:** 10.3389/fvets.2021.671460

**Published:** 2021-05-07

**Authors:** Mitchell V. Palmer, Tyler C. Thacker, Carly Kanipe, Paola M. Boggiatto

**Affiliations:** ^1^Bacterial Diseases of Livestock Research Unit, National Animal Disease Center, Agricultural Research Service, United States Department of Agriculture, Ames, IA, United States; ^2^National Veterinary Services Laboratories, Animal and Plant Health Inspection Service, Ames, IA, United States; ^3^Immunobiology Graduate Program, College of Veterinary Medicine, Iowa State University, Ames, IA, United States

**Keywords:** cytokine, granuloma, *in situ* hybridization, *Mycobacterium bovis*, pathology, tuberculosis

## Abstract

*Mycobacterium bovis* is the cause of tuberculosis in most animals, most notably cattle. The stereotypical lesion of bovine tuberculosis is the granuloma; a distinct morphological lesion where host and pathogen interact and disease outcome (i.e., dissemination, confinement, or resolution) is determined. Accordingly, it is critical to understand host-pathogen interactions at the granuloma level. Host-pathogen interactions within individual granulomas at different stages of disease have not been examined in cattle. We examined bacterial burden and cytokine expression in individual pulmonary granulomas from steers at 30, 90, 180, and 270 days after experimental aerosol infection with *M. bovis*. Bacterial burdens within individual granulomas examined 30 days after infection were greater and more heterogenous (variable) than those examined 90 to 270 days after infection. Bacterial burdens did not correlate with expression of IFN-γ, TNF-α, TGF-β, granuloma stage, or lung lesion score, although there was a modest positive correlation with IL-10 expression. Granuloma stage did have modest positive and negative correlations with TNF-α and IL-10, respectively. Heterogeneity and mean expression of IFN-γ, IL-10 and TNF-α did not differ significantly over time, however, expression of TGF-β at 90 days was significantly greater than that seen at 30 days after infection.

## Introduction

Bacteria of the genus *Mycobacterium* are Gram-positive, acid-fast bacilli. Among the many mycobacterial species, several are important human and animal pathogens such as *Mycobacterium tuberculosis* and *Mycobacterium bovis* (1). Tuberculosis in humans is primarily caused by *M. tuberculosis*; however, the zoonotic pathogen, *M. bovis*, which most commonly affects cattle can also produce tuberculosis in humans (2). The *M. tuberculosis* (*M. tb.)* complex includes *M. tuberculosis, M. bovis, M. africanum, M. microti, M. caprae, M. canetii, M. pinnipedii, M. orygis, M. suricattae, M. mun*gi, the dassie bacillus and the chimpanzee bacillus (3–9). Of all these species, *M. bovis* has the broadest host range, which includes most mammalian species, most notably cattle [bovine tuberculosis, (bTB)]. The hallmark lesion of tuberculosis, regardless of host or tissue, is the granuloma. A granuloma resulting from infection with a member of the *M. tb*. complex may also be referred to as a tuberculoid granuloma (10).

The tuberculoid granuloma is a morphologically distinctive microscopic lesion dominated by a modified epithelial-like (epithelioid) macrophage, with lesser numbers of lymphocytes and multinucleated giant cells. In humans and animals, granulomas represent the host-pathogen interface where disease outcome (i.e., dissemination, confinement, or resolution) is determined; therefore, it is critical to understand host-pathogen interactions at the granuloma level (11–13). The granuloma paradox, however, is that although it is crucial for controlling and containing infection, it also contributes to early proliferation of bacilli (14–17).

The pathogenesis of bovine tuberculosis recapitulates many aspects of human tuberculosis; indeed, cattle have been used a model of human tuberculosis (18, 19). In humans and cattle, tiny residues, <5 μm, of evaporated droplets (droplet nuclei) can be generated by an infected host while coughing, or in the case of humans, talking or singing (20–22). Such nuclei remain airborne for hours, and once inhaled can be deposited in the terminal bronchioles and alveoli. Resident alveolar macrophages phagocytose the bacilli, which in turn induces the production of cytokines, chemokines and enzymes. Expression of both pro- (e.g., IFN-γ, TNF-α,) and anti-inflammatory (e.g., IL-10, TGF-β) cytokines activate innate immune cells such as neutrophils, monocytes, macrophages and dendritic cells (14, 23). A coordinated exchange of cells between the lung and draining lymph nodes ensues as dendritic cells containing bacilli migrate from the lung to the lymph nodes. There, naïve T cells are activated through antigen presentation. Activated T cells home to the site of infection and cytokine and chemokine signals attract and activate monocytes and macrophages. As cells accumulate, the granuloma forms. The typical tuberculoid granuloma has a central region of caseous necrosis, surrounded by a zone of epithelioid macrophages and multinucleated giant cells, with the outermost zone containing increasing numbers of lymphocytes and occasional plasma cells. As infection persists, a partial to complete fibrous capsule may be produced. In addition to typical granuloma morphology, granulomas may be non-necrotizing or highly mineralized.

In cattle the heterogenous nature of bovine tuberculoid granulomas has been described in terms of morphology, as well as cellular composition and cytokine expression (24–27). Comparisons have also been made between tuberculoid granulomas arising in different organs from the same animal (25, 27). What has been less studied are granuloma changes over the course of disease, from early to late infection, including the bacterial burden within individual granulomas. Research in non-human primate (NHP) models of human tuberculosis reveals that individual granulomas are microenvironments in which local immune responses drive the bacterial burden, disease progression, and overall disease status (12, 28) indicative of disease control at the granuloma level.

The objective of the current study was to describe individual granuloma changes, in terms of morphologic stages, bacterial burdens and cytokine composition at increasing intervals after experimental aerosol infection. We hypothesized that granuloma heterogeneity may be reflected not only in morphology, but also in bacterial burden, and the concomitant cytokine environment.

## Materials and Methods

### *Mycobacterium bovis* Aerosol Challenge

*Mycobacterium bovis* strain 10-7428 was used in this experiment. This field strain was of low passage (<3) and had been shown previously to be virulent in the calf aerosol model (29). Inoculum was prepared using standard techniques (30) in Middlebrook's 7H9 liquid media (Becton Dickinson, Franklin Lakes, NJ, USA) supplemented with 10% oleic acid-albumin-dextrose complex (OADC; Difco, Detroit, MI, USA) plus 0.05% Tween 80 (Sigma Chemical Co., St. Louis, MO, USA). Mid log-phase growth bacilli were pelleted by centrifugation at 750 X g, washed twice with phosphate buffered saline (PBS) (0.01 M, pH 7.2) and stored at−80^0^ C until used. Frozen stock was warmed to room temperature and diluted to the appropriate cell density in 2 ml of PBS. Bacilli were enumerated by serial dilution plate counting on Middlebrook's 7H11 selective media (Becton Dickinson). A single dose was determined to be 1.12 X 10^4^ CFU per steer.

Aerosol infection of steers with virulent *M. bovis* has been described in detail previously (24, 29, 31). Briefly, 20 Holstein steers (9 months of age) were obtained from a source with no history of *M. bovis* infection. Steers were infected with a single dose of virulent *M. bovis* strain 10-7428 by nebulization of inoculum into a mask (Equine AeroMask®, Trudell Medical International, London, ON, Canada) covering the nostrils and mouth. All experimental animal procedures were conducted in accordance with recommendations in the Care and Use of Laboratory Animals of the National Institutes of Health and the Guide for the Care and Use of Agricultural Animals in Research and Teaching (32, 33). Animal-related procedures were also approved by the USDA-National Animal Disease Center Animal Care and Use Committee.

### Sample Collection

Five steers each were euthanized at 30, 90, 180, and 270 days after infection. Steers were humanely euthanized by intravenous administration of sodium pentobarbital. Tissues were examined for gross lesions and processed for microscopic analysis as described previously (29). Each lung lobe was examined separately and sectioned at 0.5 – 1.0 cm intervals. Lesions suspected to be granulomas were dissected out and processed as individual samples. In cases where numerous granulomas were present, a maximum of 5 granulomas each from five lung lobes were collected for a total of 25 granulomas per steer.

Individual granulomas were divided with one half being processed for mycobacterial isolation, as described below, and the other half processed for microscopic evaluation by formalin fixation. Individual granulomas and other tissue samples (≤0.5 cm in width) were fixed by immersion in 10% neutral buffered formalin (≥ 20 volumes fixative to 1 volume tissue) for approx. 24 h and transferred to 70% ethanol, followed by standard paraffin embedding techniques. Paraffin embedded samples were cut in 4 μm thick sections, transferred to Superfrost Plus™ charged microscope slides (Thermo Fisher Scientific, Pittsburg, PA) and stained with hematoxylin and eosin (H&E). For all individual granulomas, adjacent sections were stained by the Ziehl-Neelsen technique for visualization of acid-fast bacilli (AFB) and numerous near adjacent unstained sections were used for *in situ* hybridization (ISH).

### Lesion Scoring

Lungs and lymph nodes (mediastinal and tracheobronchial) were evaluated using a semiquantitative gross pathology scoring system described previously (34). Lung lobes (left cranial, left caudal, right cranial, right caudal, middle and accessory) were assessed individually based on the following scoring system: 0, no visible lesions; 1, no external gross lesions, but lesions seen on slicing; 2, <5 gross lesions of <10 mm in diameter; 3, >5 gross lesions of <10 mm in diameter; 4, >1 distinct gross lesion of >10 mm in diameter; 5, gross coalescing lesions. Cumulative mean scores were then calculated for each entire lung.

### Granuloma Bacterial Burden

Quantitative assessment of mycobacterial burden of individual granulomas was evaluated as described elsewhere (35). Only granulomas from the right and left caudal lobes, for a total of 10 granulomas per steer, were processed. Briefly, granulomas were homogenized in phenol red nutrient broth using a blender. Logarithmic dilutions (10^0^-10^9^) of homogenates in PBS were plated in 100 ml aliquots on Middlebrook 7H11 selective agar plates (Becton Dickinson) and incubated for 8 weeks at 37 °C. Isolates were confirmed to be *M. bovis* by IS6110 real time PCR as described elsewhere (36).

### Microscopic Examination

All individual granulomas were staged according to criteria described previously (24, 25, 37, 38). Stage I (initial) granulomas were characterized by accumulations of epithelioid macrophages admixed with low numbers of lymphocytes and neutrophils. Multinucleated giant cells were sometimes present, but necrosis was absent. When present, AFB were seen within macrophages or multinucleated giant cells. Stage II (solid) granulomas were characterized by accumulations of epithelioid macrophages surrounded by a thin, incomplete fibrous capsule. Infiltrates of neutrophils, lymphocytes and multinucleated giant cells were sometimes present. Necrosis was minimal to mild and centrally located. When present, AFB were seen within macrophages or multinucleated giant cells. Stage III (necrotic) granulomas were characterized by necrotic cores, some with small foci of dystrophic mineralization, surrounded by a zone of epithelioid macrophages admixed with multinucleated giant cells and lymphocytes. As distance from the necrotic core increased, the relative number of lymphocytes also increased and the number of epithelioid macrophages and multinucleated giant cells decreased. The entire granuloma was surrounded by a thin to moderate fibrous capsule. When present, AFB were seen within the necrotic core and, to a lesser extent, within macrophages and multinucleated giant cells. Stage IV (necrotic and mineralized) granulomas were characterized by a variably thick fibrous capsule surrounding irregular multicentric granulomas with multiple necrotic cores, often with foci of dystrophic mineralization. Epithelioid macrophages and multinucleated giant cells surrounded necrotic areas; these cellular infiltrates were bordered by a zone of large numbers of lymphocytes. When present, AFB were seen most often within the necrotic core.

### mRNA Chromogenic ISH

Only granulomas from the right and left caudal lobes, for a total of 10 granulomas per steer, were processed for ISH. ISH was performed on individual granulomas from 30, 90- and 270-days post-infection. RNAscope® ZZ probe technology (Advanced Cell Diagnostics, Newark, CA) was used to perform mRNA ISH in formalin-fixed paraffin-embedded (FFPE) tissue sections using the RNAscope® 2.5 HD Reagents – RED kit (Advanced Cell Diagnostics) on samples from 30, 90 and 270 days after infection. Proprietary ZZ probes complementary to mRNA sequences of interest were used for visualization of mRNA transcripts for the following cytokines: IFN-γ (Cat. No. 315581), TNF-α, (Cat. No. 316151), TGF-β (Cat. No. 427271) and IL-10 (Cat. No. 420941). A positive control probe targeted the *Bos taurus*-specific *cyclophilin B* (*PPIB*) housekeeping gene (Cat. No. 319451), while a probe targeting *dapB* of *Bacillus subtilis* (Cat. No. 310043) was used as a negative control. The RNAscope® labeling technique has been shown to be capable of single mRNA molecule detection (39).

Formalin-fixed paraffin embedded tissue pretreatment was performed with manual antigen retrieval according to the manufacturer's instructions. Slides were baked in a dry oven for 1 hr at 60°C to promote tissue-to-slide adherence, deparaffinized and rehydrated in fresh xylenes and 100% ethanol, and air dried. RNAscope® hydrogen peroxide (Advanced Cell Diagnostics) was next applied to each tissue section for 10 min at room temperature (RT, 20^0^ C) to block endogenous peroxidase activity, followed by rinsing with fresh distilled water (dH_2_O). Disruption of formalin cross-linking and unmasking of antigenic epitopes was achieved by submerging slides in a boiling 1X RNAscope® target retrieval solution (Advanced Cell Diagnostics) for 15 min, followed by rinsing with fresh dH_2_O and 100% ethanol. Once slides had completely air dried, a hydrophobic barrier was drawn around each tissue using an ImmEdge® pen (Vector Laboratories, Burlingame, CA), and slides were stored at RT overnight with desiccants. The following day, RNAscope® Protease Plus was applied to each tissue section and incubated in a humidifying tray at 40°C in a HybEZ™ Hybridization System oven (Advanced Cell Diagnostics) for 30 min. Slides were then rinsed with fresh dH_2_O before proceeding to probe hybridization.

Probe hybridization, amplification, and detection were performed according to manufacturer's instructions. All incubations were carried out in a humidifying tray either at RT or in a HybEZ™ oven at 40°C. Between each incubation step, slides were washed with fresh 1X Wash Buffer (Advanced Cell Diagnostics). To allow binding of the ZZ probe to target mRNA, customized probe prewarmed to 40°C was applied to each tissue section and incubated at 40°C for 2 hrs. Branched amplification and detection of the probe with Fast Red chromogen (Advanced Cell Diagnostics) was achieved by incubating slides with kit reagents (Advanced Cell Diagnostics) as follows: AMP 1 (30 min), AMP 2 (15 min), AMP 3 (30 min), and AMP 4 (15 min) at 40°C; AMP 5 (30 min) and AMP 6 (15 min) at RT; and a 60:1 solution of RED-A: RED-B (Advanced Cell Diagnostics) at RT for 10 min.

Following RED detection, slides were rinsed with fresh dH_2_O before being transferred to a 1:1 Gill's hematoxylin I:dH_2_O (American MasterTech, Lodi, CA) counterstain. Slides were submerged in hematoxylin solution for 2 min, rinsed with fresh dH_2_O thrice, submerged in 0.02% ammonia water for bluing, and dry baked at 40°C for 20 min or until completely dry. Tissue dehydration was not completed due to the alcohol-sensitive nature of the Fast Red chromogen. To mount the tissue samples, slides were dipped in fresh xylenes, 1–2 drops of aqueous EcoMount mounting medium (Biocare Medical, Pacheco, CA) was applied to each tissue section, and a #1 thickness cover slip was applied over top of the tissue section. Slides were dried at RT in the dark overnight before microscopic examination.

### Morphometry

Slides were scanned at 40X magnification and digitized using the Aperio ScanScope XT workstation (Aperio Technology, Inc., Vista, CA, USA). Digitized images of individual granulomas were analyzed using image analysis software (HALO™, Indica Labs, Inc., Corrales, NM). Using the RNA ISH HALO™ module, the chromogenic reaction of Fast Red was identified and the signal (i.e., red dots) quantified. The RNA ISH module algorithm identifies both cells and signal allowing the quantification of signal number per area occupied by cells within the granuloma (i.e., signal/total cell area). The entire section of each granuloma was analyzed.

### Statistical Analysis

Mean values for lesion scores, tissue weights and cytokine expression at each time point were evaluated using one-way analysis of variance, followed by Tukey's *post-hoc* multiple comparison test (GraphPad Prism 8.0, GraphPad Software, San Diego, CA, USA) to compare differences between means. Correlations between multiple variables were analyzed using Pearson correlation coefficients (GraphPad Prism 8.0). For all analyses a *p*-value < 0.05 was considered significant.

## Results

### Gross Lesions, Lesion Scores and Granuloma Stages

**Figure 1:**
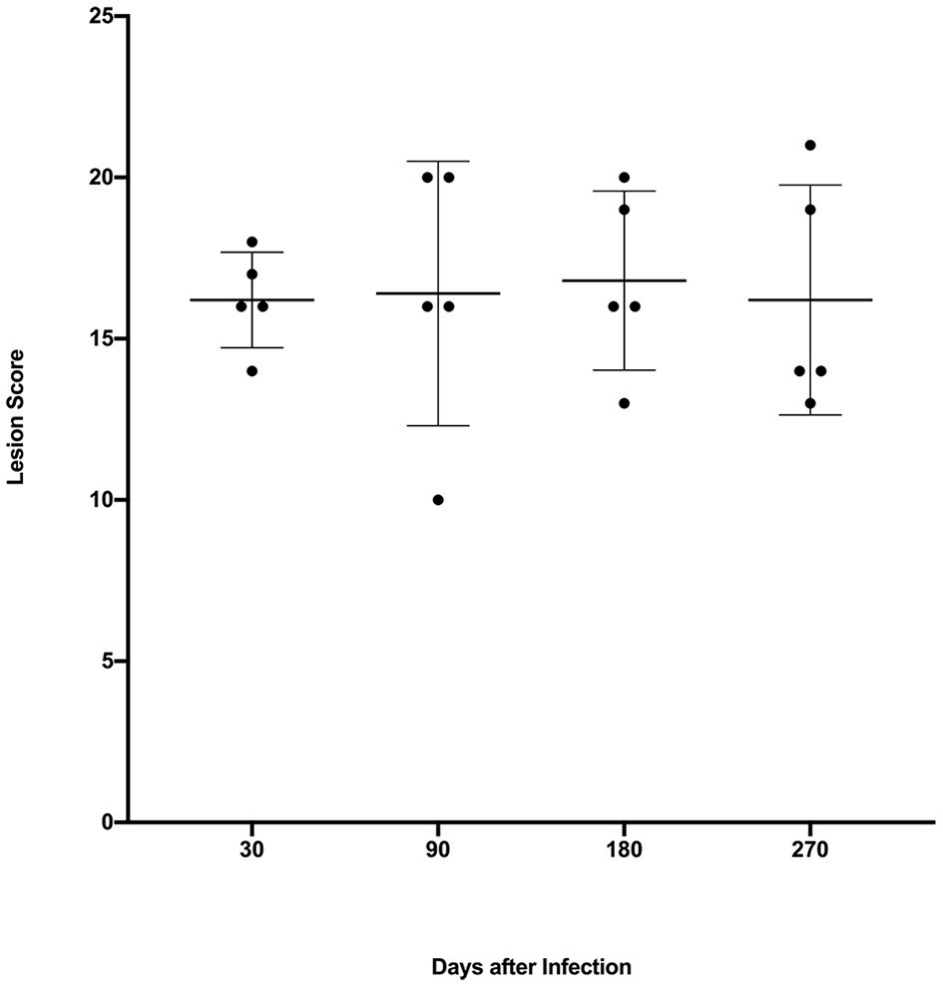
Lung lesion scores from calves experimentally infected with *M. bovis* and examined at various time points after infection. Values are expressed as individual scores with mean ± SD.

**Figure 2:**
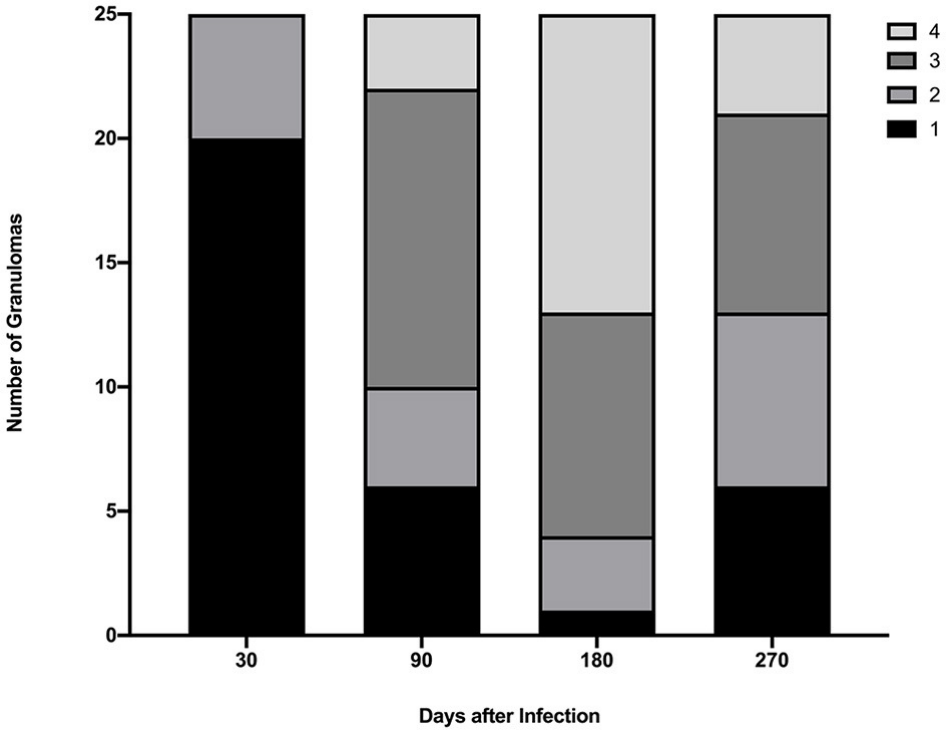
Granuloma stages collected from lungs of steers euthanized 30, 90, 180, and 270 days after experimental infection with *M. bovis*. A maximum of 5 granulomas were collected from each of 5 lung lobes (right cranial, left cranial, right caudal and middle, left caudal, and accessory).

Gross lesions were seen in lungs of all infected steers at all time points. The spread and variability (i.e., SD) of lung lesion scores within groups was similar and the means did not differ significantly (Figure 1). Granulomas examined at 30 days post-infection were predominantly stage I (Figure 2). At 90, 180- and 270-days post-infection all 4 granuloma stages were represented, albeit to varying degrees (Figure 2).

### Bacterial Burden of Individual Granulomas

**Figure 3:**
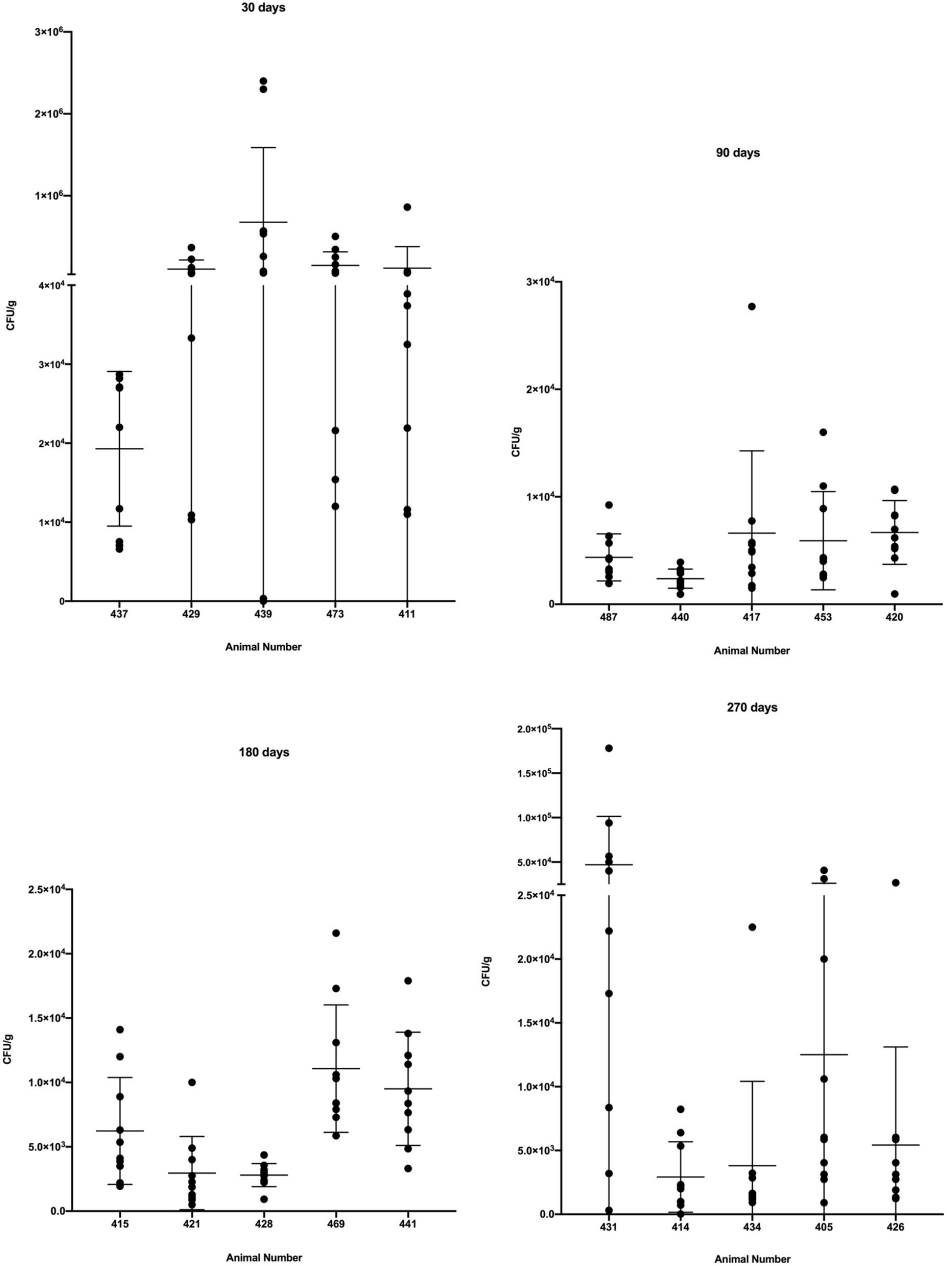
Bacterial burden in colony-forming units/g (CFU/g) in individual granulomas from steers experimentally infected with *M. bovis* and examined at 30, 90, 180, and 270 days after infection. Values represent CFU/g within individual granulomas from right and left caudal lobes with individual animal means ± SD.

As a group, steers examined 30 days after infection had a significantly higher bacterial burden (*p* < 0.0001) than cattle examined at 90, 180 and 270 days after infection (Figure 3). Additionally, as groups, the bacterial burdens of cattle examined 90, 180 and 270 days after infection did not differ significantly from each other (Figure 3). Examination of individual granulomas revealed the bacterial burdens of granulomas in all animals at all time points were heterogenous with varying degrees of dispersion. The burden range was high, as a number of granulomas from animals examined 30 days post-infection had bacterial burdens >10^5^ CFU/g while a single granuloma from steer #439, 30 days post-infection contained no culturable *M. bovis* (i.e., sterile granuloma). Although no additional sterile granulomas were noted, a single granuloma with <100 CFU/g was seen in steer #414 at 270 days post-infection.

### *In situ* Hybridization

**Figure 4:**
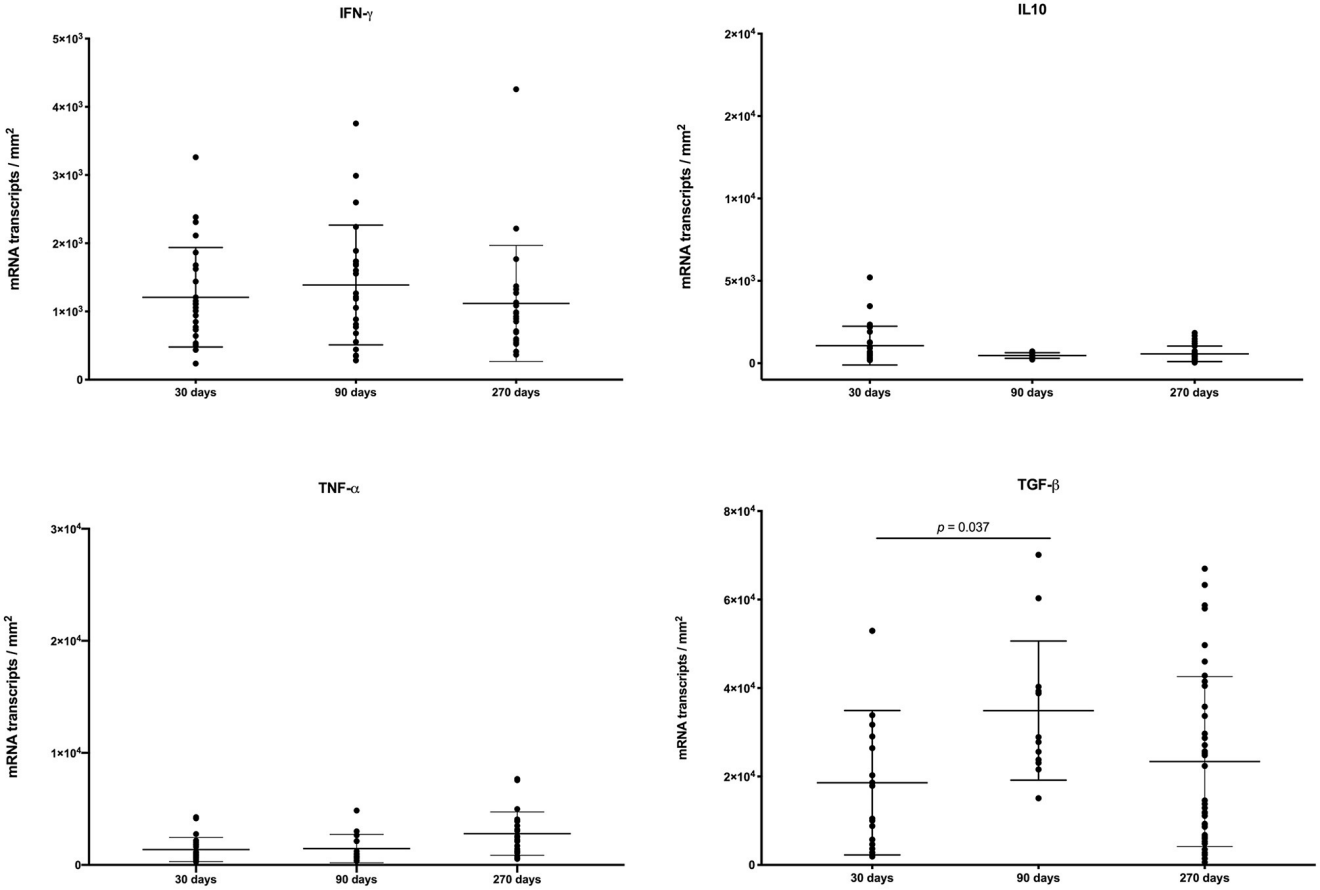
Expression of IFN-γ, IL-10, TNF-α, and TGF-β, as measured by *in-situ* hybridization in individual granulomas from right and left caudal lung lobes of steers experimentally infected with *M. bovis* and examined at 30, 90, and 270 days after infection. Values represent mRNA transcripts/mm^2^ with group means ± SD.

The balance of pro- and anti-inflammatory cytokines likely contribute to the dynamic nature of granulomas over the course of infection (40). We assessed the expression of various cytokines within granulomas from different time points after infection. Individual granuloma cytokine expression was heterogenous and the variability was greater for IFN-γ and TGF-β compared to IL-10 and TNF-α (Figure 4). However, we observed no significant differences in mean expression of IFN-γ, IL-10 or TNF-α at any time points examined, with the exception of TGF-β where expression was significantly greater at 90 days compared to 30 days.

### Correlations

When all timepoints were combined, there a was marginally positive correlation between bacterial burden and IL-10 expression; otherwise at any individual time point there was no correlation between bacterial burden and gene expression for any cytokine (Table 1). Granuloma stage was marginally positive correlated with TNF-α, and negatively correlated with IL-10 gene expression when all time-points were combined; however, no correlation was seen at individual time points (Supplementary Tables 1–3). No correlation was noted between lung lesion score and bacterial burden at any time point.

**Table 1:** Pearson correlation coefficients (r) for cytokine expression, bacterial burden (CFU/g) and granuloma stage in pulmonary granulomas examined at all time-points combined (30, 90, 180, and 270 days after infection) from calves experimentally infected with aerosolized *M. bovis*.

	**IFN-γ**	**IL-10**	**TNF-α**	**TGF-β**	**CFU/g**
IL-10	0.35^a^	–	–	–	–
	**0.007**^b^				
TNF-α	0.26	0.03	–	–	–
	**0.043**	0.790			
TGF-β	0.079	-0.042	−0.127	–	–
	0.556	0.757	0.346		
CFU/g	−0.085	0.250	−0.148	−0.055	–
	0.484	**0.032**	0.248	0.676	
Granuloma Stage	−0.04	−0.35	0.36	0.203	−0.129
	0.714	**0.003**	**0.003**	0.121	0.138

a *Pearson coefficient (r)*.

b *p-value (<0.05 are highlighted in bold text)*.

Cytokine gene expression using all time-points combined showed marginal positive correlations between IFN-γ and both IL-10 and TNF-α (Table 1). These marginal positive correlations were also seen at 30 days post-infection (Supplementary Table 1), however, only the positive correlation between IFN-γ and TNF-α carried over to the 90-day post-infection time point (Supplementary Table 2). No correlations between cytokine gene expression were noted at 270 days post-infection (Supplementary Table 3).

## Discussion

Prior studies in cattle have described the morphological heterogeneity of tuberculoid granulomas in both experimental and natural infections (26, 37, 41, 42). The present study examined bacterial burdens of individual pulmonary tuberculoid granulomas at 30, 90, 180 and 270 days after experimental aerosol infection and revealed heterogeneity in bacterial burdens at all time points. Heterogeneity of both morphology and bacterial burdens of individual granulomas has not been examined in cattle. Such heterogeneity suggests that bovine pulmonary tuberculoid granulomas may have their own behavior and trajectory as has been demonstrated in NHP models of human tuberculosis (12, 28). In spite of this independence, as a group the bacterial burden at 30 days was significantly greater than later time points. A decrease in bacterial burden beyond 30 days after infection is consistent with previous studies in cattle demonstrating evidence of a delayed type hypersensitivity (DTH) response to *M. bovis* antigens 3–4 weeks after infection (43–46). The present data would suggest that after the time required for a DTH adaptive immune response to develop there is an overall 1-2 log decrease in bacterial burdens. Nevertheless, there was only a single granuloma from which no *M. bovis* was isolated. Such sterile granulomas are not uncommon in NHP models of human tuberculosis (11, 12, 40). The NHP model has also demonstrated a higher bacterial burden in lesions arising at earlier time points and a significant reduction in bacterial burden after the onset of adaptive immunity, consistent with the findings in this study (12).

Previous studies of tuberculoid granulomas of cattle have suggested that bacterial burdens are increased in late stage IV granulomas, while others have found no correlation between bacterial numbers and granuloma stage (26, 41). Those studies, however, estimated bacterial burden by enumerating AFB observed in microscopic tissue sections. In the current study, we saw no correlation between bacterial burden and granuloma stage and there was not an observable increase in bacterial burdens in granulomas collected at later time points. To the contrary, we found bacterial burdens higher at 30 days post-infection than at later time points. Bacterial burdens did not correlate with expression of IFN-γ, TNF-α, TGF-β, granuloma stage, or lung lesion score, although there was a modest positive correlation with IL-10 expression. Granuloma stage did have modest positive and negative correlations with TNF-α and IL-10, respectively.

Considered a key cytokine, IFN-γ is critical to macrophage activation and pivotal in a protective Th1 response to mycobacteria. Therefore, an interesting finding of the present study was the steady state of IFN-γ expression over time, which did not correlate with either bacterial burden or granuloma stage. Prior studies in cattle have produced inconclusive results in terms of IFN-γ expression in granulomas. Some of these differences may be in technique, inoculation dose, duration of disease, or tissue (i.e., lymph nodes vs. lung). Previous cattle studies using IHC to measure IFN-γ protein within granulomas showed no difference in expression based on granuloma stage; however, there was increased IFN-γ expression in calves receiving a higher dose (1,000 CFU vs. 1 CFU) (42). A separate study using IHC showed an increase in IFN-γ protein immunolabeling between stages I and IV (41). Yet, another study, also using IHC, showed IFN-γ in granulomas of all stages, with a qualitative, but not significant decrease in labeling in late stage IV granulomas compared to earlier stages (47). A study using mRNA extracted from formalin-fixed paraffin-embedded lymph node cross sections showed no significant differences in expression levels of IFN-γ, TNF-α or IL-10 at 5, 12, and 19 weeks after infection and no correlation between lesion severity and individual cytokine expression (48). Supporting the present findings, a study using ISH methods similar to those used here, demonstrated that IFN-γ expression did not differ between stage I and stage IV granulomas examined at a single time point (150 days) after experimental infection (24).

In contrast to IFN-γ expression, there was a mild, but not significant trend of increasing TNF-α gene expression from 30 to 270-days post-infection. Increasing TNF-α levels, may be associated with the increased presence of necrosis within granulomas as they progress from stage I, non-necrotizing granulomas to stage IV granulomas with significant necrosis. Necrosis is a consistent feature of many tuberculoid granulomas, regardless of mycobacterial species, host species or target organ, intimating the importance of this process in the pathogenesis of tuberculosis. Tissue destruction facilitates spread of disease as bacilli gain entrance to vascular elements, which carry them to distant sites. Other studies using granulomas from cattle collected at a single time-point after experimental infection, or from naturally infected cattle of unknown disease duration have shown decreasing trends, increasing trends, or no difference, respectively, between TNF-α expression in granulomas of stage I to IV. (24, 41, 47, 49).

In tuberculosis, TNF-α has been shown to be critical, with low levels associated with fatal disease; however, excessive TNF-α induces a hyper-inflammatory environment (50, 51). TNF-α induces cytokine production by macrophages, activates macrophages for killing, and modulates macrophage apoptosis (52). Considered pro-inflammatory, cytokines such as TNF-α and IFN-γ if left uncontrolled can lead to excessive tissue damage, increased inflammation, and deteriorating disease status. To balance inflammation and tissue destruction, IL-10 deactivates macrophages, resulting in diminished Th1 cytokine production and decreased production of reactive nitrogen and oxygen species (53). The present study suggests that early in infection, when bacterial burdens are higher and inflammation is increasing, IL-10 production is stimulated. This stimulation appears less critical at later time points when bacterial burdens are lower. In mice, overexpression of IL-10 is associated with increased bacterial burdens, while in humans with tuberculosis, elevated levels of IL-10 have been associated with more active disease and more severe clinical signs (54, 55).

Examinations of TGF-β expression in granulomas from experimentally infected cattle have yielded conflicting results. In one study of lymph node granulomas from experimentally infected cattle examined 29 weeks after infection TGF-β expression was greater in more advanced granulomas compared to early stage granulomas (37). Two other studies found lower expression in late stage granulomas compared to early stage granulomas (49, 56). TGF-β is a key cytokine implicated in fibrinogenesis and new collagen synthesis. In macaques, TGF-β has been suggested as a critical driver of tissue repair in active tuberculosis (57). As more advanced granulomas are often characterized by a peripheral fibrous capsule, the presence of increased TGF-β expression at 90 days compared to 30 days, as seen in the present study, is consistent with a role in fibrosis.

In NHPs positron emission tomography-computed tomography (PET-CT) imaging has allowed serial imaging during the course of infection producing a dynamic map of individual granuloma activity that can be correlated with postmortem exam findings (11, 12). The present study in cattle attempted to follow the course of infection by examining individual animals at increasing intervals after infection. This method yields multiple snap shots of granuloma activity at defined time points, but does not allow observation of individual granuloma development, activity and disease progression within an animal. Thus, a shortcoming of the present study is that we are unaware of the state of individual granulomas before or after the defined time points. Nevertheless, examination of individual pulmonary granulomas of cattle did reveal significant heterogeneity in bacterial burdens, however it is less clear how bacterial burdens relate to granuloma development or cytokine production within granulomas.

## Data Availability Statement

The raw data supporting the conclusions of this article will be made available by the authors, without undue reservation.

## Ethics Statement

The animal study was reviewed and approved by USDA-National Animal Disease Center Animal Care and Use Committee.

## Author Contributions

MP and TT: experiment design. MP and CK: sample collection and experiments. MP and PB data analysis. MP: manuscript preparation. MP, TT, CK, and PB: manuscript editing. All authors contributed to the article and approved the submitted version.

## Conflict of Interest

The authors declare that the research was conducted in the absence of any commercial or financial relationships that could be construed as a potential conflict of interest.

## References

[1] Sizemore C Lacourciere K Parker T. The many hosts of mycobacteria: an interdisciplinary approach to understanding mycobacterial diseases. In: Mukundan H Chambers MA Waters RW Larsen MH, editors. Tuberculosis, Leprosy and Mycobacterial Diseases of Man and Animals. Boston, MA: CABI. (2015).

[2] Langer A LoBue PA. Public health significance of zoonotic tuberculosis in animals and humans. In: Thoen CO Steele JH Kaneene JB, editors. Zoonotic Tuberculosis: Mycobacterium bovis and other Pathogenic Mycobacteria. Ames: Wiley Blackwell. (2014) p. 21–34.

[3] Rodriguez-Campos S Smith NH Boniotti MB Aranaz A. Overview and phylogeny of *Mycobacterium tuberculosis* complex organisms: implications for diagnostics and legislation of bovine tuberculosis. Res Vet Sci. (2014) 97:S5–19. 10.1016/j.rvsc.2014.02.009 24630673

[4] Coscolla M Gagneux S. Consequences of genomic diversity in *Mycobacterium tuberculosis*. Semin Immunol. (2014) 26:431–44. 10.1016/j.smim.2014.09.012 25453224 PMC4314449

[5] Cousins DV Bastida R Cataldi A Quse V Redrobe S Dow S. Tuberculosis in seals caused by a novel member of the *Mycobacterium tuberculosis* complex: *Mycobacterium pinnipedii* sp. nov. Int J Syst Evol Microbiol. (2003) 53:1305–14. 10.1099/ijs.0.02401-0 13130011

[6] Cousins DV Francis BR Gow BL Collins DM McGlashan CH Gregory A. Tuberculosis in captive seals: bacteriological studies on an isolate belonging to the *Mycobacterium tuberculosis* complex. Res Vet Sci. (1990) 48:196–200. 10.1016/S0034-5288(18)30990-1 2110376

[7] Cousins DV Peet RL Gaynor WT Williams SN Gow BL. Tuberculosis in imported hyrax (*Procavia capensis*) caused by an unusual variant belonging to the *Mycobacterium tuberculosis* complex. Vet Microbiol. (1994) 42:135–45. 10.1016/0378-1135(94)90013-2 7886928

[8] Parsons SD Drewe JA Gey van Pittius NC Warren RM van Helden PD. Novel cause of tuberculosis in meerkats, South Africa. Emerg Infect Dis. (2013) 19:2004–7. 10.3201/eid1912.130268 24274183 PMC3840885

[9] Alexander KA Laver PN Michel AL Williams M van Helden PD Warren RM. Novel *Mycobacterium tuberculosis* complex pathogen, *M*. Mungi. Emerg Infect Dis. (2010) 16:1296–9. 10.3201/eid1608.100314 20678329 PMC3298296

[10] Myers RK McGavin MD Zachary JF. Cellular adaptations, injury, and death: Morphologic, biochemical, and genetic bases. In: Zachary JF McGavin MD, editors. Pathologic Basis of Veterinary Disease. St. Louis: Elsevier Mosby. (2012) p. 122–4.

[11] Lin PL Coleman T Carney JP Lopresti BJ Tomko J Fillmore D. Radiologic responses in cynomolgous macaques for assessing tuberculosis chemotherapy regimens. Antimicrob Agents Chemother. (2013) 57:4237–44. 10.1128/AAC.00277-13 23796926 PMC3754323

[12] Lin PL Ford CB Coleman MT Myers AJ Gawande R Ioerger T. Sterilization of granulomas is common in active and latent tuberculosis despite within-host variability in bacterial killing. Nat Med. (2014) 20:75–9. 10.1038/nm.3412 24336248 PMC3947310

[13] Gideon HP Skinner JA Baldwin N Flynn JL Lin PL. Early whole blood transcriptional signatures are associated with severity of lung inflammation in cynomolgus macaques with *Mycobacterium tuberculosis* infection. J Immunol. (2016) 197:4817–28. 10.4049/jimmunol.1601138 27837110 PMC5289749

[14] Cicchese JM Evans S Hult C Joslyn LR Wessler T Millar JA. Dynamic balance of pro- and anti-inflammatory signals controls disease and limits pathology. Immunol Rev. (2018) 285:147–67. 10.1111/imr.12671 30129209 PMC6292442

[15] Davis JM Ramakrishnan L. The role of the granuloma in expansion and dissemination of early tuberculous infection. Cell. (2009) 136:37–49. 10.1016/j.cell.2008.11.014 19135887 PMC3134310

[16] Ramakrishnan L. Revisiting the role of the granuloma in tuberculosis. Nat Rev Immunol. (2012) 12:352–66. 10.1038/nri3211 22517424

[17] Flynn JL. Mutual attraction: does it benefit the host or the bug? Nat Immunol. (2004) 5:778–9. 10.1038/ni0804-778 15282559

[18] Waters WR Palmer MV. *Mycobacterium bovis* infection of cattle and white-tailed deer: translational research of relevance to human tuberculosis. ILAR J. (2015) 56:26–43. 10.1093/ilar/ilv001 25991696

[19] Waters WR Maggioli MF McGill JL Lyashchenko KP Palmer MV. Relevance of bovine tuberculosis research to the understanding of human disease: historical perspectives, approaches, and immunologic mechanisms. Vet Immunol Immunopathol. (2014) 159:113–32. 10.1016/j.vetimm.2014.02.009 24636301

[20] Wells WF Ratcliffe HL Grumb C. On the mechanics of droplet nuclei infection; quantitative experimental air-borne tuberculosis in rabbits. Am J Hyg. (1948) 47:11–28. 18921435 10.1093/oxfordjournals.aje.a119179

[21] Loudon RG Roberts RM. Droplet expulsion from the respiratory tract. Am Rev Respir Dis. (1967) 95:435–42. 6018703 10.1164/arrd.1967.95.3.435

[22] Loudon RG Roberts RM. Singing and the dissemination of tuberculosis. Am Rev Respir Dis. (1968) 98:297–300. 5667756 10.1164/arrd.1968.98.2.297

[23] Etna MP Giacomini E Severa M Coccia EM. Pro- and anti-inflammatory cytokines in TB: a two-edged sword in TB pathogenesis. Semin Immunol. (2014) 26:543–51. 10.1016/j.smim.2014.09.011 25453229

[24] Palmer MV Thacker TC Waters WR. Analysis of cytokine gene expression using a novel chromogenic in-situ hybridization method in pulmonary granulomas of cattle infected experimentally by aerosolized *Mycobacterium bovis*. J Comp Pathol. (2015) 153:150–9. 10.1016/j.jcpa.2015.06.004 26189773

[25] Palmer MV Thacker TC Waters WR. Differential cytokine gene expression in granulomas from lungs and lymph nodes of cattle experimentally infected with aerosolized *Mycobacterium bovis*. PLoS ONE. (2016) 11:e0167471. 10.1371/journal.pone.0167471 27902779 PMC5130274

[26] Palmer MV Waters WR Thacker TC. Lesion development and immunohistochemical changes in granulomas from cattle experimentally infected with *Mycobacterium bovis*. Vet Pathol. (2007) 44:863–74. 10.1354/vp.44-6-863 18039899

[27] Shu D Heiser A Wedlock DN Luo D de Lisle GW Buddle BM. Comparison of gene expression of immune mediators in lung and pulmonary lymph node granulomas from cattle experimentally infected with *Mycobacterium bovis*. Vet Immunol Immunopathol. (2014) 160:81–9. 10.1016/j.vetimm.2014.03.017 24852075

[28] Cadena AM Fortune SM Flynn JL. Heterogeneity in tuberculosis. Nat Rev Immunol. (2017) 17:691–702. 10.1038/nri.2017.69 28736436 PMC6247113

[29] Waters WR Thacker TC Nelson JT DiCarlo DM Maggioli MF Greenwald R. Virulence of two strains of *Mycobacterium bovis* in cattle following aerosol infection. J Comp Pathol. (2014) 151:410–9. 10.1016/j.jcpa.2014.08.007 25306158

[30] Larsen MH Biermann K Jacobs WR Jr. Laboratory maintenance of *Mycobacterium tuberculosis*. Curr Protoc Microbiol. (2007) 6:10A.1.1–10A.1.8. 10.1002/9780471729259.mc10a01s6 18770602

[31] Palmer MV Waters WR Whipple DL. Aerosol delivery of virulent *Mycobacterium bovis* to cattle. Tuberculosis. (2002) 82:275–82. 10.1054/tube.2002.0341 12623270

[32] Garber JC. Guide for the Care and Use of Laboratory Animals. Washington, DC: The National Academies Press (2011) 1–246. 21595115

[33] Federation of Animal Science Societies. Guide for the Care and Use of Agricultural Animals in Research and Teaching. Champaign, IL: Federation of Animal Science Societies (2010).

[34] Vordermeier HM Chambers MA Cockle PJ Whelan AO Simmons J Hewinson RG. Correlation of ESAT-6-specific gamma interferon production with pathology in cattle following *Mycobacterium bovis* BCG vaccination against experimental bovine tuberculosis. Infect Immun. (2002) 70:3026–32. 10.1128/IAI.70.6.3026-3032.2002 12010994 PMC128013

[35] Waters WR Palmer MV Nonnecke BJ Thacker TC Scherer CF Estes DM. Failure of a *Mycobacterium tuberculosis* DeltaRD1 DeltapanCD double deletion mutant in a neonatal calf aerosol *M*. bovis challenge model: comparisons to responses elicited by M. bovis bacille calmette guerin. Vaccine. (2007) 25:7832–40. 10.1016/j.vaccine.2007.08.029 17931755

[36] Thacker TC Harris B Palmer MV Waters WR. Improved specificity for detection of *Mycobacterium bovis* in fresh tissues using IS6110 real-time PCR. BMC Vet Res. (2011) 7:50. 10.1186/1746-6148-7-50 21867516 PMC3170578

[37] Wangoo A Johnson L Gough J Ackbar R Inglut S Hicks D. Advanced granulomatous lesions in *Mycobacterium bovis*-infected cattle are associated with increased expression of type I procollagen, gamma delta (WC1+) T cells and CD 68+ cells. J Comp Pathol. (2005) 133:223–34. 10.1016/j.jcpa.2005.05.001 16154140

[38] Johnson L Gough J Spencer Y Hewinson G Vordermeier M Wangoo A. Immunohistochemical markers augment evaluation of vaccine efficacy and disease severity in bacillus Calmette-Guerin (BCG) vaccinated cattle challenged with *Mycobacterium bovis*. Vet Immunol Immunopathol. (2006) 111:219–29. 10.1016/j.vetimm.2006.01.016 16540176

[39] Wang F Flanagan J Su N Wang LC Bui S Nielson A. RNAscope^?^: a novel *in situ* RNA analysis platform for formalin-fixed, paraffin-embedded tissues. J Mol Diagn. (2012) 14:22–9. 10.1016/j.jmoldx.2011.08.002 22166544 PMC3338343

[40] Gideon HP Phuah J Myers AJ Bryson BD Rodgers MA Coleman MT. Variability in tuberculosis granuloma T cell responses exists, but a balance of pro- and anti-inflammatory cytokines is associated with sterilization. PLoS Pathog. (2015) 11:e1004603. 10.1371/journal.ppat.1004603 25611466 PMC4303275

[41] Tulu B Martineau HM Zewude A Desta F Jolliffe DA Abebe M. Cellular and cytokine responses in granulomas of asymptomatic cattle naturally infected with *Mycobacterium bovis* in Ethiopia. Infect Immun. (2020) 88:e00507–20. 10.1128/IAI.00507-20 32958527 PMC7671892

[42] Johnson L Dean G Rhodes S Hewinson G Vordermeier M Wangoo A. Low-dose *Mycobacterium bovis* infection in cattle results in pathology indistinguishable from that of high-dose infection. Tuberculosis. (2007) 87:71–6. 10.1016/j.tube.2006.04.002 16723276

[43] Waters WR Palmer MV Nonnecke BJ Thacker TC Scherer CF Estes DM. Efficacy and immunogenicity of *Mycobacterium bovis* DeltaRD1 against aerosol *M*. bovis infection in neonatal calves. Vaccine. (2009) 27:1201–9. 10.1016/j.vaccine.2008.12.018 19135497 PMC2750035

[44] Dean GS Rhodes SG Coad M Whelan AO Cockle PJ Clifford DJ. Minimum infective dose of *Mycobacterium bovis* in cattle. Infect Immun. (2005) 73:6467–71. 10.1128/IAI.73.10.6467-6471.2005 16177318 PMC1230957

[45] Rhodes SG Palmer N Graham SP Bianco AE Hewinson RG Vordermeier HM. Distinct response kinetics of gamma interferon and interleukin-4 in bovine tuberculosis. Infect Immun. (2000) 68:5393–400. 10.1128/IAI.68.9.5393-5400.2000 10948169 PMC101803

[46] Thom ML Hope JC McAulay M Villarreal-Ramos B Coffey TJ Stephens S. The effect of tuberculin testing on the development of cell-mediated immune responses during *Mycobacterium bovis* infection. Vet Immunol Immunopathol. (2006) 114:25–36. 10.1016/j.vetimm.2006.07.001 16904754

[47] Aranday-Cortes E Bull NC Villarreal-Ramos B Gough J Hicks D Ortiz-Pelaez A. Upregulation of IL-17A, CXCL9 and CXCL10 in early-stage granulomas induced by *Mycobacterium bovis* in cattle. Transbound Emerg Dis. (2013) 60:525–37. 10.1111/j.1865-1682.2012.01370.x 22909117

[48] Witchell J Maddipatla SV Wangoo A Vordermeier M Goyal M. Time dependent expression of cytokines in *Mycobacterium bovis* infected cattle lymph nodes. Vet Immunol Immunopathol. (2010) 138:79–84. 10.1016/j.vetimm.2010.07.004 20696483

[49] Aranday-Cortes E Hogarth PJ Kaveh DA Whelan AO Villarreal-Ramos B Lalvani A. Transcriptional profiling of disease-induced host responses in bovine tuberculosis and the identification of potential diagnostic biomarkers. PLoS ONE. (2012) 7:e30626. 10.1371/journal.pone.0030626 22359547 PMC3281027

[50] Dorhoi A Kaufmann SH. Tumor necrosis factor alpha in mycobacterial infection. Semin Immunol. (2014) 26:203–9. 10.1016/j.smim.2014.04.003 24819298

[51] Flynn JL Goldstein MM Chan J Triebold KJ Pfeffer K Lowenstein CJ. Tumor necrosis factor-alpha is required in the protective immune response against *Mycobacterium tuberculosis* in mice. Immunity. (1995) 2:561–72. 10.1016/1074-7613(95)90001-2 7540941

[52] Flynn JL Chan J Lin PL. Macrophages and control of granulomatous inflammation in tuberculosis. Mucosal Immunol. (2011) 4:271–8. 10.1038/mi.2011.14 21430653 PMC3311958

[53] Beamer GL Flaherty DK Assogba BD Stromberg P Gonzalez-Juarrero M de Waal Malefyt R. Interleukin-10 promotes *Mycobacterium tuberculosis* disease progression in CBA/J mice. J Immunol. (2008) 181:5545–50. 10.4049/jimmunol.181.8.5545 18832712 PMC2728584

[54] Bonecini-Almeida MG Ho JL Boechat N Huard RC Chitale S Doo H. Down-modulation of lung immune responses by interleukin-10 and transforming growth factor beta (TGF-beta) and analysis of TGF-beta receptors I and II in active tuberculosis. Infect Immun. (2004) 72:2628–34. 10.1128/IAI.72.5.2628-2634.2004 15102771 PMC387880

[55] Verbon A Juffermans NP van Deventer SJ Speelman P VanDeutekom HV. Serum concentrations of cytokines in patients with active tuberculosis (TB) and after treatment. Clin Exp Immunol. (1999) 115:110–3. 10.1046/j.1365-2249.1999.00783.x 9933428 PMC1905191

[56] Salguero FJ Gibson S Garcia-Jimenez W Gough J Strickland TS Vordermeier HM. Differential cell composition and cytokine expression within lymph node granulomas from BCG-vaccinated and non-vaccinated cattle experimentally infected with *Mycobacterium bovis*. Transbound Emerg Dis. (2017) 64:1734–49. 10.1111/tbed.12561 27615603

[57] DiFazio RM Mattila JT Klein EC Cirrincione LR Howard M Wong EA. Active transforming growth factor-beta is associated with phenotypic changes in granulomas after drug treatment in pulmonary tuberculosis. Fibrogenesis Tissue Repair. (2016) 9:6. 10.1186/s13069-016-0043-3 27148404 PMC4855369

