# Supplementary material for: Heterogeneity of Pulmonary Granulomas in Cattle Experimentally Infected With Mycobacterium bovis
Source: Front Vet Sci. 2021 May 7;8:671460. doi: 10.3389/fvets.2021.671460 (PMC8138452; doi:10.3389/fvets.2021.671460)
Supplement: Supplementary file 1 [file Table_1.docx]

**Supplementary Table 1**. Pearson correlation coefficients (r) for cytokine expression, bacterial burden (CFU/g) and granuloma stage in pulmonary granulomas collected 30 days after infection from calves experimentally infected with aerosolized *M. bovis*.

|  | IFN-γ | IL-10 | TNF-α | TGF-β | CFU/g |
| --- | --- | --- | --- | --- | --- |
| IL-10 | 0.52^1^  **0.008**^2^ | -- | -- | -- | -- |
| TNF-α | 0.59  **0.002** | 0.31  0.142 | -- | -- | -- |
| TGF-β | 0.20  0.33 | 0.39  0.06 | 0.56  **0.004** | -- | -- |
| CFU/g | 0.019  0.926 | -0.006  0.979 | 0.165  0.442 | 0.187  0.370 | -- |
| Granuloma Stage | 0.03  0.902 | -0.29  0.214 | -0.30  0.162 | -0.43  **0.031** | -0.116  0.581 |

^1^ Pearson coefficient (r).

^2^ *p*-value (<0.05 are highlighted in bold text).
