# Supplementary material for: Heterogeneity of Pulmonary Granulomas in Cattle Experimentally Infected With Mycobacterium bovis
Source: Front Vet Sci. 2021 May 7;8:671460. doi: 10.3389/fvets.2021.671460 (PMC8138452; doi:10.3389/fvets.2021.671460)
Supplement: Supplementary file 2 [file Table_2.docx]

**Supplementary Table 2**. Pearson correlation coefficients (r) for cytokine expression, bacterial burden (CFU/g) and granuloma stage in pulmonary granulomas collected 90 days after infection from calves experimentally infected with aerosolized *M. bovis*.

|  | IFN-γ | IL-10 | TNF-α | TGF-β | CFU/g |
| --- | --- | --- | --- | --- | --- |
| IL-10 | 0.63^1^  0.094^2^ | -- | -- | -- | -- |
| TNF-α | 0.64  **0.034** | -0.15  0.634 | -- | -- | -- |
| TGF-β | 0.28  0.410 | 0.26  0.439 | -0.25  0.382 | -- | -- |
| CFU/g | 0.112  0.649 | 0.053  0.870 | -0.160  0.570 | 0.218  0.435 | -- |
| Granuloma Stage | 0.07  0.782 | -0.06  0.848 | 0.49  0.067 | 0.155  0.580 | 0.076  0.719 |

^1^ Pearson coefficient (r).

^2^ *p*-value (<0.05 are highlighted in bold text).
