# Supplementary material for: Heterogeneity of Pulmonary Granulomas in Cattle Experimentally Infected With Mycobacterium bovis
Source: Front Vet Sci. 2021 May 7;8:671460. doi: 10.3389/fvets.2021.671460 (PMC8138452; doi:10.3389/fvets.2021.671460)
Supplement: Supplementary file 3 [file Table_3.docx]

**Supplementary Table 3.** Pearson correlation coefficients (r) for cytokine expression, bacterial burden (CFU/g) and granuloma stage in pulmonary granulomas collected 270 days after infection from calves experimentally infected with aerosolized *M. bovis*.

|  | IFN-γ | IL-10 | TNF-α | TGF-β | CFU/g |
| --- | --- | --- | --- | --- | --- |
| IL-10 | 0.01^1^  0.989^2^ |  |  |  |  |
| TNF-α | 0.08  0.746 | 0.32  0.346 |  |  |  |
| TGF-β | -0.012  0.962 | 0.537  0.170 | 0.858  2.7 x 10^-6^ |  |  |
| CFU/g | -0.125  0.610 | -0.080  0.724 | 0.496  **0.019** | 0.422  0.072 |  |
| Granuloma Stage | -0.33  0.162 | -0.31  0.168 | 0.37  0.086 | 0.327  0.160 | 0.391  **0.017** |

^1^ Pearson coefficient (r).

^2^ *p*-value (<0.05 are highlighted in bold text).
